# Antiviral effect of Evusheld in COVID-19 hospitalized patients infected with pre-omicron or omicron variants: a modelling analysis of the randomized DisCoVeRy trial—authors’ response

**DOI:** 10.1093/jac/dkae447

**Published:** 2025-01-13

**Authors:** Maxime Beaulieu, Jérémie Guedj

**Affiliations:** IAME, INSERM, Université Paris Cité, Paris F-75018, France; IAME, INSERM, Université Paris Cité, Paris F-75018, France

We thank Lacout *et al*.^1^ for their comments on our study,^2^ and would like to respectfully suggest several possible misinterpretations of our data and results.

(a, b) Lacout *et al*. seem to overlook three decades of research on viral dynamics that has led to dozens of publications which provided major insights into the quantitative understanding of viral infections.^3–6^ These models are considered as ‘semi-mechanistic’ as they build on host–pathogen interaction to propose a mathematical framework of analysis. In fact, Lacout *et al*. probably confuse our approach with machine learning models that are biology agnostic and indeed require large amounts of information, as exemplified by their citation of Baker *et al*., which refers to machine learning models and not (semi)-mechanistic models. One of the advantages of biology-based models is precisely that parameters have a biological meaning, which allows the fixing of parameters based on prior knowledge (see Methods section in the original article). From a statistical standpoint, our model was not used to fit each individual separately, and Lacout *et al*. perhaps did not understand that we used a mixed-effect model,^7^ a statistical approach that borrows strength from the whole sample to estimate parameter distribution in a population. Table S2 (in Supplementary data of the original article) indicates that the model estimates 19 parameters (fixed effects or variance parameters), which are obtained by simultaneously fitting all virological and immunological data, i.e. 633 viral load measurements and 485 neutralization measures collected in 199 individuals. Note that, unlike what Lacout *et al*. imply, our model accounts for vaccination status [see second paragraph of the Results and Table S2 (in original article)] and differential protection across individuals against viral infection.

(c–e) Lacout *et al*. also more generally question the use of antiviral drugs in COVID-19. Lacout *et al*. seem to ignore the high efficacy of mAbs as pre-exposure prophylaxis (PreP) or in outpatients, including Evusheld (AZD-7442),^8^ which has led to its authorization as PreP in high-risk patients in several countries, including France.^9^ Although it is obvious that antiviral strategies are most efficient when given early after infection,^10^ our study aimed to explore the possibility that hospitalized patients may also benefit from antiviral treatments, as clearly discussed in the first paragraph of the article. In a previous study we demonstrated that Evusheld was safe and well tolerated, showing no excess cardiac events.^11^ However, Evusheld did not show clinical efficacy, which may be due to the fact that treatment was administered too late and/or to the insufficient statistical power due to premature trial interruption. Thus, the identification of a virological benefit is one element that may suggest that antiviral strategies could still be beneficial, at least for some individuals with high viral load, as an additional weapon in the arsenal against COVID-19. Note that in the DisCoVeRy trial, all patients received the standard of care, which included oxygen supplementation and anti-inflammatory drugs.^11^

(f) Lastly, the discussion on remdesivir is irrelevant to our study, but we point Lacout *et al*. to the exhaustive analysis of adverse events observed in the first part of the DisCoVeRy trial that addressed remdesivir evaluation.^12^

To conclude, our study identified an antiviral effect of Evusheld in hospitalized patients. Whether this translates into a clinically meaningful benefit is not known, but warrants the evaluation of mAbs with high neutralization activity and long-lasting activity in COVID-19 hospitalized patients and potentially in other viral respiratory infections.
